# Perception of Urban Public Safety of Floating Population with Higher Education Background: Evidence from Urban China

**DOI:** 10.3390/ijerph17228663

**Published:** 2020-11-22

**Authors:** Ju He, Yunxiao Dang, Wenzhong Zhang, Li Chen

**Affiliations:** 1 Key Laboratory of Regional Sustainable Development Modeling, Institute of Geographic Sciences and Natural Resources Research, CAS, Beijing 100101, China; hej.19b@igsnrr.ac.cn; 2 University of Chinese Academy of Sciences, Beijing 100039, China; 3 Land and Urban-Rural Development Research Institute, Zhejiang University of Finance and Economics, Hangzhou 310018, China; dangyx.09s@igsnrr.ac.cn; 4 College of Applied Arts and Science, Beijing Union University, Beijing 100191, China; chenli@buu.edu.cn

**Keywords:** urban public safety, floating population, HLM, influencing factor, China

## Abstract

The Corona Virus Disease 2019 (COVID-19) outbreak caused people to pay significant attention to urban public safety issues. The city’s public safety is an important part of the high-quality development and the construction of a liveable city. To understand whether and how factors at different levels affect the public security of particular group of people in a city. This study uses data from an extensive questionnaire survey by the Ministry of Housing and Urban-Rural Development of the People’s Republic of China (MOHURD) in 11 cities. This study uses the descriptive statistical method and Hierarchical Linear Model (HLM) to study the perception of urban public safety (PUPS) and its influencing factors of floating population with higher education background (FPHEB) from the three levels of city–district–individual. The study finds that (1) when FPHEB is placed in a district and a city at the same time, the influence of the city on PUPS is greater than that of the district; (2) the urban’s infrastructure security and economic development security positively affect the floating population; (3) the GDP and the number of stadiums and hospitals of the district are significantly positively correlated with the PUPS of the FPHEB, whereas the increase of population density and road density have negative effects; (4) FPHEB with distinct attributes will make their PUPS also different. This study is not only a reflection on the construction of urban public security after the COVID-19 outbreak but can also be used as a theoretical reference for the government in constructing urban public security. This study also enriches the research on the floating population and makes good scientific suggestions for the city’s PUPS of the FPHEB. The research results can provide a better reference for the government’s urban safety construction from the perspective of residents’ perception.

## 1. Introduction

Safety is the foundation of sustainable urban development. In the process of urban development, public health security incidents, natural disasters and daily social security incidents have occurred frequently, which poses a challenge to urban safety construction [1]. In accelerating urbanisation, emergency facilities and organisational capabilities of cities when responding to safety incidents are critical in the context of increasing population mobility [2]. From the end of 2019, the Corona Virus Disease 2019 (COVID-19) epidemic spread globally. It has caused the government and the public to attach great importance to urban safety and rethink it. Under the people-oriented urban planning and construction concept, how to evaluate urban safety, especially the safety evaluation based on the residents’ perspective, is very important to reflect on urban safety construction.

Urban public security is the premise that the city can ensure the development of social economy and infrastructure construction of the city [3]. In the face of man-made (i.e., traffic accidents, building fire disasters, etc.) and natural disasters (i.e., earthquakes, floods and other weather related disasters, etc.), people have a safe refuge space and adequate medical facilities [4,5,6]. In the process of urban development, people become the core of urban development, and cities begin to focus on the quality of life [7,8,9,10]. Therefore, urban public security also considers good ecological environment, residents’ living environment and residents’ own conditions. The perception of public safety is closely related to the environment in which people live. Therefore, to improve people’s sense of urban security, the government is committed to the construction of urban public safety, which will reduce the losses caused by security incidents. The current quantitative research on public safety focuses on analysing the temporal and spatial distribution of public safety incidents [11,12] and the configuration of disaster prevention facilities [13,14]. A balanced construction of regional security resilience is necessary, and this epidemic has exposed this problem. From a meso-scale perspective, the emergency response efficiency and the number of medical facilities in each district in Wuhan vary. From a macro-scale perspective, due to differences in economic levels and supporting facilities, different provinces and cities have various epidemic control effects [15]. We can see that the safety construction of different city levels is different. The city scale is constructed by the government from the overall plan and goals, and the district scale is the management and implementation of specific safety construction. Therefore, the construction of resilient cities must promote the balanced development of various scales and regions and improve overall resilience [16,17]. Many studies exist on evaluating urban security resilience from an objective perspective [18,19,20]. However, studies that explore the security and resilience between cities and at all levels within cities from the perspective of the public’s subjective perception remain limited. Therefore, whether different levels of security construction will have different effects on people’s perception of security. This is very meaningful for the government’s decision-making on safety construction.

Given the differences in individual sensitivity and ability to respond to disasters, residents of different attributes have distinct perceptions of public safety. Scholars have begun to conduct research on generalized “safety perception” involving public health safety, emergency safety and natural disaster safety. For the people, safety itself is the most basic need and the most vital interest. Pei-Yi found that in general hospital emergencies, men’s sense of security is low [21]. Hamama-Raz Y.(2015) studied the characteristics of the population affected by Sandy Zhaofeng in the United States and uncovered that women are more concerned about potential disaster risks than men after disasters, but no significant difference exists between the two sexes regarding sources of support [22]. Graif found that mothers of low-income and ethnic minorities felt socially uneasy and isolated in terms of organisation after Hurricane Katrina [23]. Thompson investigated people who may be at great risk from bush fires. The elderly, families with young children, frail and self-identified persons with disabilities will be greatly affected in terms of safety [16]. Theory of social vulnerability defines these groups who are more vulnerable in security incidents as vulnerable groups and points out that low-income groups, the elderly, ethnic minorities and other physically and socially vulnerable groups are more vulnerable than other groups [24] and these people tend to have lower safety ratings. In addition, studies pointed out that when individuals evaluate the city’s sense of security, their trust in others and other emotional sustenance will also have an impact [25].

In the context of China’s urbanisation, the floating population has become an important part of Chinese cities [26,27]. They play a pivotal role in the process of urbanization [28]. For the floating population, the household registration system became an institutional barrier for their survival and development in the city [29]. Without a local hukou, they are not eligible to enjoy many local social benefits and services, including minimum living allowance and subsidised housing. The floating population will live in different environments because of their income, occupation, etc. Many migrants live in low-cost communities [30,31] and poor living environments are often accompanied by relatively high crime rates [32,33]. Therefore, living environment may cause the floating population to be sensitive to their perception of public safety in different cities. In addition, the characteristics of the migrant population, especially factors such as gender, age, income, occupation and family characteristics, will affect their satisfaction with the city or their willingness to move [30,33,34]. It can be seen that the existing research has confirmed that the personal attributes of the floating population have an impact on the sense of security. The research conclusions are also very similar. However, there is no in-depth exploration of the special population among the floating population. This article selects the higher education group among the floating population as the research object. This group is very special. They have high education and high income. Many of them own real estate in cities where they live. Their lives are stable, and their willingness to settle in the city is high, which is the main force for urbanization and the implementation of population urbanization. Therefore, it is important to pay attention to their feelings.

Urban security is the foundation of urban development and the basic guarantee for residents’ long-term survival. The floating population with higher education qualifications (FPHEQ) is the main force in the implementation of population urbanization. It is important to understand their perception of urban public safety. Whether and how different levels of the city affect them. These all have important guiding for government in decision-making. For this purpose, based on a review of existing research, this study mainly discusses the perception of urban public safety (PUPS) of floating population with higher education background (FPHEB) from the “city–district–individual” levels. We used nearly 10,000 questionnaires which were taken in 11 cities and their 73 districts by the Ministry of Housing and urban-rural development of the People’s Republic of China (MOHURD) in 2019. We constructed an indicator system of influencing factors of safety perception from three levels and used a Hierarchical Linear Model (HLM) to explore their inner relationships. Through this study, we can understand how different urban backgrounds affect the PUPS of the FPHEQ.

Therefore, it can be seen that this article focuses on the PUPS of floating population with higher education qualifications, and also, it explores the detailed factors affecting the public safety of the city, including which levels’ factors. Such research is of great significance to the government’s decision-making on safe city construction. We hope to answer the following questions:

① What are the differences in the perception of urban public safety of FPHEQ between cities and districts within the city?
② How different groups of FPHEQ in the city perceive urban public safety?
③ Will the different levels of the city have an impact on the public security of the city and which level has a greater impact on the safety perception of FPHEQ?

## 2. Research Design

### 2.1. Indicator Framework Construction

Built environment, social environment and individual characteristics are the three major factors that affect residents’ sense of security [35]. This article focuses on the impact of these three factors on residents’ sense of security from three levels: city, district and individual. Due to the different functions of different city levels, their impact on residents’ safety perception is also very different. In addition, as the definition of urban public safety above. The factors themselves are different. Infrastructure is the basic guarantee of urban public security, economic development is the driving force, and natural ecological environment is the quality improvement of urban safe life. Only in this way can the security of the whole social development be guaranteed. Differences at different levels are also inevitable. District level is the space where people most often move, and it is also the concrete implementation of urban public security.

**Figure 1:**
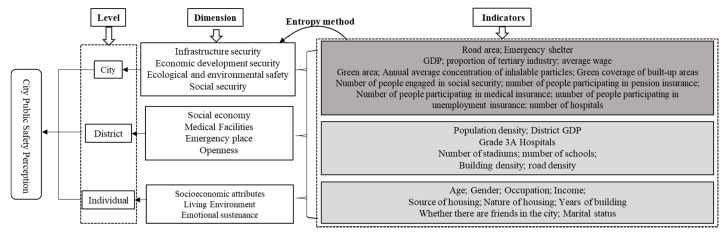
Indicator system framework of “city–district–individual”.

As the macro-scale in the urban system, the city is the target carrier for the government to carry out safety planning and development. According to the existing research on the construction of the index system of urban safety evaluation, urban safety is mainly embodied in four aspects: infrastructure safety, economic development safety, ecological environment safety and social security [36,37]. Existing research shows that the factors of the city have a certain influence on the residents’ perception of the city [38,39,40]. Therefore, this study assumes that city-level factors will affect people’s perception of safety from those four dimensions, and we selected indicators based on these four dimensions. Moreover, we used the Entropy method to calculate the value of each dimension as an explanatory variable at the city level (Figure 1).

District level, as a mesoscale, is the specific implementation of urban safety construction. At present, the research on subjective perception of city has relatively abundant research from the micro- (streets, communities) and mesoscale (cities) [40,41,42], while the research results from the meso-micro (district) are relatively few. Whether district-level factors will affect the residents’ PUPS has not been well-established literature reference. The district level is the spatial scope of people’s long-term stable activities in the city. Existing studies have found that hospitals, sports facilities [43], recreational facilities [44], building density and road density [45] in the built environment have a sensitive impact on the sense of security. The population density [45,46] and economic level [47] in the social environment will also affect people’s perception of safety. During the epidemic, schools and stadiums were used as shelter hospitals to treat patients in Wuhan and other cities. In addition, schools and stadiums are also important places to face other urban security incidents. Based on the above, this article selects district-level indicators from four aspects: Social economy, Medical facilities, Emergency place and Openness. After collinearity check, we used seven indicators from these four aspects.

At the individual level, research has pointed out that the sensitivity of different groups of people to security incidents and their response capabilities vary with their personal characteristics. Vulnerable groups such as children, the elderly, women and low-income families are most likely to be harmed by emergency security incidents [48]. In addition, social capital, migrants, ownership of housing property rights and source of housing will also affect personal security perception [35,44]. In terms of the living environment, the nature of the housing will lead to differences in its supporting safety facilities, property management service level, community openness and overall quality of residents. In terms of housing sources, if it is a leased house, the mobility of the residents themselves and their neighbourhoods will increase, and the strangeness of the surrounding environment may reduce their sense of security. However, the renters have a more tolerant security choice space for the living environment, which may also improve their sense of security [35]. The age of the building will bring about new and old problems. Buildings that have been built for a long time will face many problems in resisting natural disasters, household water and electricity and self-repair, which may bring a lower sense of security to residents. The presence or absence of friends or partners in the city will have a greater impact on floating population’s emotional sustenance, which may affect their sense of security. This paper selects FPHEB which is a special group of the floating population as research object, so the personal attribute index excludes samples such as low education, local household registration, under 18 years of age, and elderly groups. Individual attributes selected age, gender, income, occupational status, housing nature and source, etc.

### 2.2. Data Source and Pre-Processing

The data in this article are mainly subjective and objective:

In terms of objective data, city-related data mainly comes from the “China City Statistical Yearbook 2018”. We selected indicators from the four dimensions of society (i.e., number of people participating in unemployment insurance; number of hospitals, etc.), economy (i.e., GDP; proportion of tertiary industry; average wage, etc.), ecology (i.e., Green area; Annual average concentration of inhalable particles, etc.) and infrastructure (i.e., Road area; Emergency shelter, etc.) After standardising all the indicators, then we used the Entropy method to calculate the comprehensive score of each dimension District-level statistical data mainly come from the statistical yearbook of each city or government website. We collected the data of Grade 3A Hospitals, schools, stadiums and so on and obtained Points of Interest (POI) through Gaode Map (https://www.amap.com/). We obtained the vector data, such as buildings and roads from the official website of OpenStreetMap (https://www.openstreetmap.org/). We verified and standardised the above data for avoiding collinearity.

**Figure 2:**
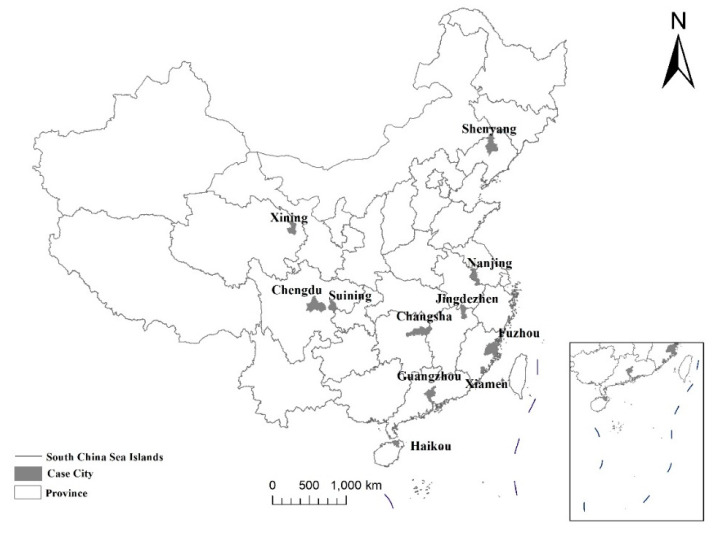
Location of the study area cities in China.

In terms of subjective data, under the organisation of the Ministry of Housing and urban-rural development of the People’s Republic of China (MOHURD), we selected 11 cities, including Shenyang, Nanjing, Fuzhou, Xiamen, Jingdezhen, Changsha, Guangzhou, Haikou, Chengdu, Suining and Xining. These 11 cities are located in various places in China. Their natural background, economic level and population size are all different. They are diverse, contrasting and representative (Figure 2). We conducted the survey by online questionnaires in the 11 cities involving 73 districts. For the questionnaire survey, we used a combination of equidistant random sampling, convenience sampling and traffic control quota sampling. The survey objects are residents who lived for over half a year. Ultimately, we distributed 13,438 online questionnaires and collected 12,050 valid questionnaires, with an effective rate of 89.7% (about 1000 sample questionnaires per city). In this survey questionnaire, we conducted the satisfaction of urban public safety using a 5-point Likert scale. From high to low, we assigned five types of “very satisfied, satisfied, average, dissatisfied and very dissatisfied” with a value of 100, 80, 60, 40 and 20 points. The research object of this paper is FPHEB. On the basis of the residence time, household registration items of the respondents [26,28] and academic qualifications, we defined those with a college degree and above and who have lived in the city for more than half a year with no local hukou as the FPHEB. After data screening and deletion of missing values, we finally obtained 4689 samples. Table 1 shows the collected statistics and descriptive statistics of questionnaire data.

### 2.3. Research Methods and Variable Settings

This study explored the PUPS of FPHEB and its influencing factors from the three levels of “city–district–individual”. These three levels have a nested relationship on the geographical background, that is, individuals are nested in urban areas and cities, and urban areas are also nested in cities. When variables have nested relationships in geographic space, compared with single-level economics, HLM can distinguish the impact of different levels on individuals and can accurately calculate the differences in individual security perceptions of different levels contributions [49].

This study took urban public safety satisfaction as the explained variable. Based on survey data, the result of satisfaction evaluation is an ordered 5-point Likert variable. Given that this study focuses on the factors that affect urban public safety and does not pay attention to the degree of safety perception, we converted the five categorical variables into binary categorical variables. If it is converted to a common continuous variable, it will not only lead to information loss, but also easily cause conversion maladjustment. We assigned 1 for the “very satisfied” and “satisfied” values. We assigned a value of 0 for the “dissatisfied” and “very dissatisfied”, and the evaluation as “general” is considered to be insensitive to residents” PUPS, thus we removed it. We explained the explanatory variables above and hence are not repeated here.

## 3. Empirical Findings

### 3.1. Inter-City and Inter-District Differences in FPHEB’s PUPS

**Figure 3:**
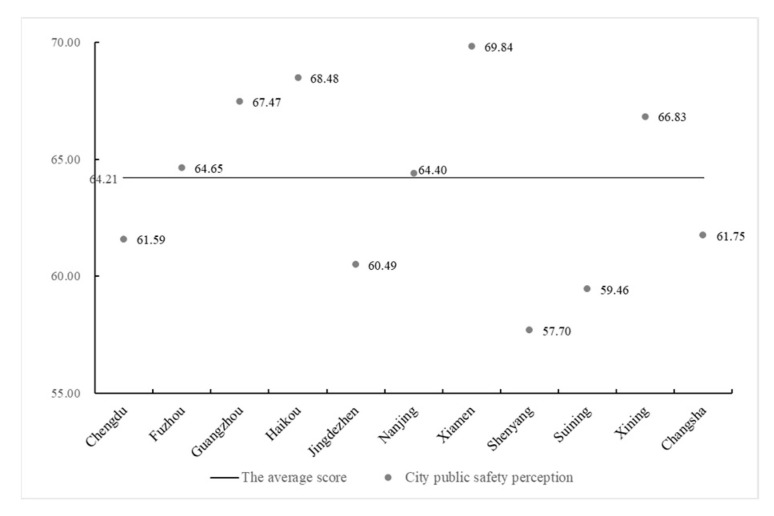
Evaluation of the floating population with higher education background (FPHEB)’s perception of urban public safety (PUPS) in different cities.

We counted the average value of migrant population’s PUPS in 11 cities (Figure 3) and found that the average value is 64.21 points. Six cities exist above the average level. Amongst them, Fuzhou and Nanjing are similar to the average. Xiamen has the highest score, followed by Haikou, Guangzhou and Xining. The other five cities have lower than average value, and Shenyang has the lowest score, followed by Suining and Jingdezhen.

**Figure 4:**
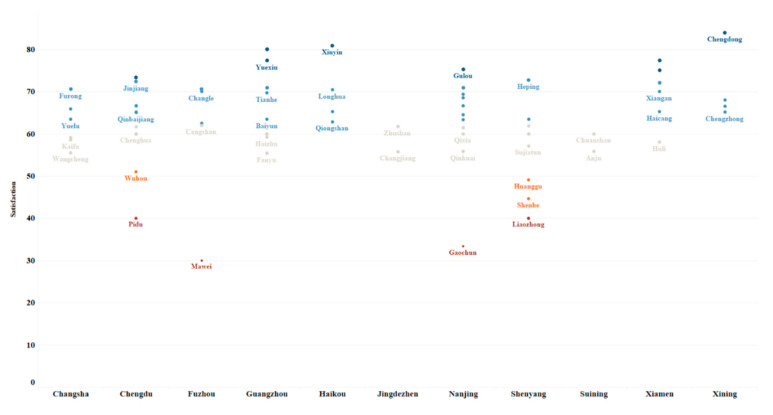
Evaluation of the FPHEB’s PUPS in different districts.

Figure 4 shows the average value of the FPHEB’s PUPS in 73 districts. The district-level public safety perception scores are basically concentrated ranging from 60–70 points. The lower levels include Mawei District in Fuzhou, Gaochun District in Nanjing and Pidu District in Chengdu. Amongst them, Mawei, Pidu and Gaochun districts are relatively remote in their respective cities, and their economic levels are relatively backwards. Districts with higher scores are Chengdong District in Xining, Xiuying District in Haikou and Huangpu and Nansha districts in Guangzhou.

### 3.2. Model Identification of Factors Affecting PUPS

We found that all three levels of factors will have an impact on the PUPS of FPHEB by the preliminary test of the model. There is a necessary condition for the use of Hierarchical Linear Model. That is, what proportion of the variance of each level displayed by the empty model (zero model), which represents the degree of influence of the factors at that level [50]. To make the model achieve the optimal effect and avoid the other levels’ factors in the perception of personal urban public safety, which may sometimes be caused by city-level or district-level factors, we constructed three zero models to analyse the influencing factors at the three levels. With the help of MlwiN2.26 (University of Bristol, Queens Road, Bristol, UK), we introduced a (Multilevel Model) Zero Model to obtain the proportions of variance at different levels. The variance of each level is greater than 0.059 (significant at the level of 0.001). That is ICC (intraclass correlation coefficient) > 0.059. On the one hand, it shows that in order to explain the difference of hierarchical factors, using HLM is necessary for the analysis [50,51]. On the other hand, we can see from the results which level has the greatest impact (Table 2). From the results of the two double-level zero models, ICC _district_ > ICC _city_, it shows that the influence of district-level factors is greater than that of city-level factors, but this is based on changes in the proportion of individual variance. From the perspective of the three-tier model (individuals are placed in both district and urban spaces at the same time), urban factors have greater effect on FPHEB’s PUPS than district factors.

### 3.3. Influence from Different Levels

#### 3.3.1. Impact of Individual Factors on the PUPS

As Table 3 shows, Model I mainly discussed the relationship between the personal socioeconomic attributes, emotional sustenance, living environment of FPHEB and their PUPS. (1) In terms of personal attributes, occupation and income are all significantly correlated with the PUPS, whereas gender and age are not. Party and government workers have a significant negative correlation with the PUPS, because such groups have higher social status, stable income and better social welfare. In terms of household income, higher income will bring them higher PUPS. (2) In terms of emotional sustenance, marital status and the presence or absence of friends in the city are significantly correlated with the PUPS. Amongst them, the married group has a significant negative correlation with the PUPS. Compared with no friends in the city, “have friends” will improve the PUPS. (3) In terms of living environment, respondents whose buildings have been built older have a lower PUPS than who live in the new buildings. As for the source of housing, compared with commercial house, living in unit houses and policy houses have a lower PUPS, whereas respondents living in self-built houses have no correlation with the PUPS.

After adding individual attribute variables into the district level variable, the correlation and significance in Models II to III are basically the same. In conclusion, those people who are “retirees, lacking friends, living unit houses or policy, living in the older buildings” have low PUPS, whereas those who “married, have friends in the cities, higher income, work in the government” have higher PUPS. In addition, the age, gender and nature of housing have no significant relationship with PUPS.

#### 3.3.2. Impact of District Factors on the PUPS

According to Table 3, Model II shows that a significant negative correlation exists between population density and PUPS, that is, the greater the urban population density, the worse the respondents’ PUPS. According to the research of criminal geography, the probability of criminal incidents will be higher in high density population gathering area [45,46]. The higher the district GDP will take, the higher the PUPS of FPHEB. The greater the density of roads in the districts, the lower the floating population’s PUPS, whereas the building density has no significant correlation with it. Areas with high density roads also lead to more traffic accidents [45]. Therefore, these high-density environments will also greatly reduce FPHEB’s PUPS. The more Grade 3A Hospitals will take, the higher PUPS of FPHEB. As we all know, hospitals are places to save lives. Therefore, the more quality hospitals around us, the safer we feel. The more sports venues will increase the respondents’ PUPS, whereas the number of schools has no significant relationship with it. This may be because people are more likely to be injured in sports, and stadiums are places where sports are concentrated. However, when there is a sudden disaster, like the COVID-19 outbreak, stadiums can be better places to take refuge. Under normal circumstances (in the absence of disaster), people may not realise it.

In Model I and Model II, the district-level variances are 0.103 and 0.089, and the inter-group variance decomposition coefficient ICC remains above 5.9%, indicating that differences in district-level factors will affect residents’ PUPS. All above, the district’s GDP, the number stadiums and the number of Grade 3A Hospitals will increase FPHEB’s PUPS. The increase in population density and road density will reduce FPHEB’s PUPS. The number of schools and the density of buildings and the number of schools have no significant relationship with it.

#### 3.3.3. Impact of City Factors on the PUPS

We constructed a three-tier model of “city–district–individual” to analyse the influencing factors of the city and district. We introduced all levels’ variables and set district level and individual level factors as control variables in the models. However, the model results are not ideal. It shows that when the model includes the factors of city and district at the same time, they will interfere with each other. Therefore, we constructed a two-tier model of “city–individual” to analyse the influencing factors of the city. Table 4 only displays the cities’ results.

Model III shows that infrastructure security and economic development security are significantly positively correlated with the FPHEB’s PUPS, whereas social security and ecological and environmental security have no relationship with it. This shows that the better the city’s infrastructure and economic development, the higher the PUPS of FPHEB with higher education degrees. From absolute values, a complete urban infrastructure will bring the population with higher education qualifications higher PUPS than the economic development security. From the absolute value of the coefficient, the coefficient at the city-level is generally greater than the district-level factor, indicating that city factors have a greater impact on PUPS.

In Model III, the Variance _city_ is 0.071, the correlation coefficient between the groups is ICC = 0.0663, the ICC _model III_ is relatively small, only slightly higher than 0.059. The correlation coefficient between the groups must be greater than 0.059 in the actual study. Using an HLM for analysis is necessary, but the correlation coefficient between groups is not an absolute “gold standard”. Muthen (1994) proposed that for research design effects, smaller correlation coefficients between groups may also must be considered [52]. Therefore, we believe that the results of Model III are valid.

Ultimately, Model III shows that city’s infrastructure security and economic development security are significantly related to PUPS, whereas the social security and ecological and environmental security have no significant correlation with it. For floating population with higher education background, just like the construction of urban public security, the security of urban infrastructure is the most important foundation, and economic security is a strong support for public security. Then, the ecological environment security and social security are both requirements for the quality of life. For the present, there is no significant relationship with them.

## 4. Discussion

The COVID-19 outbreak reflects the weakness of the city when facing public safety incidents, and the public paid unprecedented attention to public safety issues. However, a lack of research on the relationship between residents’ public safety perception and external environment in the fields of geography and urban planning persists. This study considers the construction of urban public safety in the context of the epidemic. We chose the floating population with higher education background to explore their PUPS and its influencing factors.

According to the research results, cities with high economic levels and complete infrastructure will bring a high PUPS to FPHEB. This will attract more floating population to the city [53,54,55]. However, we need to anticipate this has entailed phenomena, such as rising wages and the acceleration of urban road construction [56,57], which has brought people greater work pressure and the probability of public traffic safety incidents [57,58]. These thereby may reduce the FPHEB’s PUPS. Therefore, in the future research, we should consider the research on the threshold of city specific indicators.

In addition, in China, the expansion of urban space usually entails an imbalance of urban and suburban development [59]. The economic level, medical facilities, road network density and other supporting facilities of the districts under the jurisdiction of the city are polarized [60,61,62]. Similar to cities, districts with high economies and complete medical facilities will give FPHEB better PUPS, and highroad density will reduce their PUPS. In addition, this study found that a district with more stadiums will bring people a high sense of urban public safety, which may be related to the possibility of being an emergency shelter [63]. At the time of the COVID-19 outbreak, many sports venues in China, especially Wuhan, acted as temporary hospitals (Fangcang shelter hospitals) and were one of the important battlefields for medical treatment [64]. This time, the stadiums had played its emergency role not only in the face of natural disasters. We believe it will bring people a higher sense of urban security in the future.

According to the results of the analysis of individual factors in this study, cities should pay more attention to buildings that were built for a long time, and they should increase the repair and even demolition of dangerous buildings to improve the ability of buildings to resist risks. The government should also provide security measures for unit houses or policy houses of the FPHEB and improve residential services to give the FPHEB a high sense of urban public security. Proceeding from self-regulation, the FPHEB wandering elsewhere should establish their own circle of friends in the urban space where they often move. They should live together with their spouses in the same city as much as possible to avoid separation in different places and improve their sense of security through emotional sustenance. In addition, cities must pay significant attention to retired people and unmarried people with no friends in the city. The research results of the impact of personal economic attributes on the PUPS are basically similar to the existing research conclusions of urban satisfaction and happiness [31,40,65]. Moreover, the results are also consistent with the research results of scholars in other countries. When encountering urban public security incidents, low-income families, elderly and single group groups have a low sense of security [22,23,24].

This study verified the relationship between the FPHEB’s PUPS and the external environment. This research is an important supplement to the study of public safety issues and also provides new content for the study of floating population. Concurrently, the research conclusions have practical application value for urban management and have positive significance for guiding urban planning, balancing the urban internal area and improving residents’ quality of life. However, this study is limited in that such a large-scale survey cannot cover all cities and districts. Nonetheless, these 11 cities and 73 districts have certain differences and their own uniqueness in terms of natural background, spatial distribution, city level and economic level. Each city is representative. This is also the criterion for us to select the questionnaire distribution area. In addition, in future studies, we should consider choosing the city where the migrant population is registered as the reference group for comparative research.

## 5. Conclusions and Recommendations

The main research conclusions are as follows: (1) In different countries, FPHEB will have the different perception of urban public safety. There are also differences between different districts. (2) City, district and individual attributes all have an impact on FPHEB’s PUPS. Individual attributes have the biggest impact. When they are in a system, the influence of cities is greater than that of districts. If individuals are placed separately in districts and cities, the influence of districts will be stronger than that of cities. (3) Cities with higher economic level and complete infrastructure will bring FPHEB a higher PUPS. (4) In districts, population density and road density will reduce the FPHEB’s PUPS. On the contrary, higher GDP, more stadiums or a larger number of hospitals will improve it.

Findings in this paper have several important policy implications. The population density of the district should not be exceedingly high and must be controlled within a reasonable range, which can improve the PUPS of the FPHEB. Gymnasiums should conduct regular facility inspections and store materials for use in the event of urban security incidents. In addition, districts should increase the number of hospitals and even hospitals dedicated to the migrant population. Cities also must develop social security institutions, increase the number of hospitals and provide better medical security environments for the FPHEB. The government should promote the establishment of an insurance protection mechanism for retired people. All these will be significant towards improving the urban public safety perception of the FPHEB. It is also key for cities to attract people with higher education and to build safe cities.

## Figures and Tables

**Table 1:** Description and statistics of selected variables.

Category	Indicator Variable	Variable Description (Mean/Percentage)	Data Sources
Explanatory variables	City public safety satisfaction		
City-level variables			
Infrastructure security	Road area (km^2^)	60.73	China 2018 City Statistical Yearbook
	Number of emergency shelters	101.18	
Economic Development security	GDP (100 million yuan)	6152.18	
	The proportion of tertiary industry	60.16	
	Average salary level (RMB)	78,515.00	
Ecological and Environmental security	Green area (hm^2^)	32,678.73	
	Annual average concentration of inhalable particles (μg/m^3^)	51.18	
	Green coverage rate of built-up area	42.67	
Social security	Number of employees in social security	59,559.73	
	Number of pension insurance participants	3,037,784.45	
	Number of participants in medical insurance	2,442,768.27	
	Number of participants in unemployment insurance	1,701,260.27	
	Number of hospitals	195.91	
District-level variables			
Social economy	Area (km^2^)	467.09	Statistical yearbook of each city; Statistical bulletin
	Population (10,000 people)	180.58	
	Population density (persons/km^2^)	5833.46	
	District GDP (100 million yuan)	864.35	
Medical facilities	Number of Grade 3A Hospitals	3.71	POI data
Emergency place	Number of stadiums	54.6	
	Number of schools	198.19	
Openness	Building density	0.014	OSM data
	Road density	3.958	
Individual-level variables			
Age	20–29 years old (29.23%); 30–39 years old (13.99%); 40–49 years old (22.52%); 50–59 years old (20.70%); 60–69 years old (13.56%);		
Gender	Male (52.33%); Female (47.67%)		
Marriage	Married (76.31%); Single (23.69%)		
Education	Junior college (39.58%); Undergraduate and above (60.42%)		
Occupation	Party and government workers (11.52%); Enterprise employees (30.76%); Self-employed persons (8.45%); Other occupation s (28.86%); Retired (20.41%)		
Income	<69,000 (18.37%); 70,000–299,000 (56.34%); 300,000–1,000,000 (16.98%); >1,000,000 (8.31%)		
Number of Friends	Very much (25.58%); Some (48.76%); A few (25.29%); no (0.36%);		
Years of building	1980 previous (9.69%); 1980–1989 (13.70%); 1990–1999 (17.20%); 2000–2009 (24.78%); 2010–now (19.17%); unclear (15.45%)		
Housing nature	Purchased (44.75%); Rented (55.25%)		
Source of housing	Commercial house (31.63%); Unit house (27.55%); Policy house (38.05%); Self-built house (2.55%); Others (0.22%)		

**Table 2:** Variance component estimates model of FPHEB’s PUPS.

Model	Individual-Level Ariance (Ratio)	District-Level Variance (Ratio)	City-Level Variance (Ratio)
Model I (Individual–district)	1 (89.53%)	0.117 (10.47%)	NA
Model II (Individual–city)	1 (93.02%)	NA	1.075 (6.98%)
Model III (Individual–district–city)	1 (79.50%)	0.095 (7.50%)	0.17 (13.44%)

**Table 3:** Results of the FPHEB’s PUPS in “district–individual” HLM.

Variable	Model I	Model II
Constant	0.662 ***	0.706 ***
District-level variables		
The population density		−0.559 **
GDP		0.223 **
Building density		−0.049
Road density		−1.058 **
Number of hospitals		0.209 **
Number of stadiums		0.438 *
Number of schools		0.223
Individual-level variables		
Age (reference group: 60–69 years old)		
20–29	−0.280	−0.286
30–39	0.320	0.319
40–49	−0.092	−0.106
50–59	−0.299	−0.321
Gender (reference group: male)		
Female	0.043	0.042
Marital status (reference group: unmarried)		
Married	1.031 ***	1.037 ***
Occupation (reference group: enterprise employees)		
Party and government workers	0.445 **	0.4432 **
Self-employed persons	−0.367	−0.377
Other occupations	0.022	0.022
Retired	−0.599 **	−0.635 **
Family income per year (reference group: >1,000,000)		
<69,000	0.438 **	0.440 **
70,000–299,000	0.856 ***	0.848 ***
30,0000–1,000,000	0.009	0.014
Number of city friends (reference group: Much)		
Some	0.462 **	0.457 **
a few	0.043	0.048
No	−0.508 **	−0.540 **
The year the house was built (reference group: Before 1980)		
1980–1989	0.510 **	0.488 **
1990–1999	0.537 **	0.530 **
1999–2009	0.523 **	0.493 **
2010–now	0.763 **	0.748 **
unclear	0.606	0.619
Housing nature (reference group: purchased)		
Rented	−0.027	−0.034
Source of housing (reference group: Commercial house)		
Unit house	−1.011 ***	−1.016 ***
Policy house	−0.440 **	−0.465 **
Self-built house	0.856	0.803
Others	−0.789	−0.755
District-level Variances	0.103	0.089
ICC	9.34%	8.17%

Notes: * significant at 10%; ** significant at 5%; *** significant at 1%.

**Table 4:** Results of the FPHEB’s PUPS in “city–individual” HLM.

City-Level Variables	Model III
Infrastructure security	7.196 **
Economic development security	3.307 **
Ecological and environmental security	−14.725
Social security	1.675
District-level variables	/
Individual-level variables	/
City-level variances	0.071
ICC	6.63%

Notes: ** significant at 5%.
